# Ontogenetic development of gonads and external sexual characters of the protandric simultaneous hermaphrodite peppermint shrimp, *Lysmata vittata* (Caridea: Hippolytidae)

**DOI:** 10.1371/journal.pone.0215406

**Published:** 2019-04-19

**Authors:** Daming Chen, Fang Liu, Zhihuang Zhu, Qi Lin, Chaoshu Zeng, Haihui Ye

**Affiliations:** 1 College of Ocean and Earth Sciences, Xiamen University, Xiamen, China; 2 Fisheries Research Institute of Fujian, Xiamen, China; 3 College of Science & Engineering, James Cook University, Townsville, Australia; Shanghai Ocean University, CHINA

## Abstract

The peppermint shrimp *Lysmata vittata* (Caridea: Hippolytidae) is a marine caridean shrimp popular in marine aquarium trade. The species is known to display the sexual system of protandric simultaneous hermaphrodite. In this study, based on captive bred specimens, the complete ontogenetic gonad development of *L*. *vittata* was studied both morphologically and histologically, from newly settled juveniles until they reached euhermaphrodite phase. It was found that in all specimens examined (carapace length: 1.8–8.5 mm), including the newly settled juveniles, possessed ovotestes, which comprised of an anterior ovarian and a posterior testicular part. Based on both morphological (e.g., size, color and shape) and histological features (e.g., oogenesis and spermatogenesis), four gonadal development stages were defined and described for *L*. *vittata*. From Stage I to III, the testicular part of the gonad became gradually mature but the ovarian part was still immature, which is defined as the male phase. At the male phase, cincinulli (5–8 hooks) presented at the tips of the appendix interna on the first pair of pleopods while appendices masculinae (AM), in a form of a stick structure with spines, presented at the inner edge of the appendix interna (AI) on the second pair of pleopods. At Stage IV, both the testicular part and the ovarian part were mature and hence is defined as euhermaphrodite phase. At the euhermaphrodite phase, most individuals lacked cincinulli and appendices masculinae on the first and second pair of pleopods respectively. This is the first time that complete ontogenetic gonadal and external sexual character development have been described and staged for a species from the genus *Lysmata* from newly settled juveniles to euhermaphrodite phase.

## Introduction

The vast majority of crustacean species are gonochoristic, however, the caridean shrimp species belonging to the family Hippolytide display more diverse sexual systems of both gonochorism and hermaphroditism. For instance, in the genus *Thor*, *T*. *manningi* is hermaphroditic while *T*. *dobkini* and *T*. *floridanus* are gonochoristic [1, 2]. In the genus Hippolyte, both *H*. *obliquimanus* and *H*. *williamsi* are reportedly gonochoristic [3, 4]. Moreover, all known species from the genus *Lysmata* have so far been confirmed as protandric simultaneous hermaphrodite (PSH) [5], i.e., the shrimp first mature as male (male phase), and then gain female function at a later stage as the shrimp grow, when it remains functional male with gonad produces both eggs and sperms simultaneously (euhermaphrodite phase).

The species from the genus *Lysmata* are distributed throughout the world and are found in tropical, subtropical and temperate waters [6]. Most of them live in coastal areas, such as reefs and gravel in intertidal zone [7], with gorgeous colors and the habit of cleaning parasites for fish [8]. In home aquaria, the shrimps can prey on and control the nuisance anemone *Aippasi apallida*, hence they are popular among aquarium hobbyists [9]. So far, the study on reproductive biology of the genus *Lysmata* has mainly focused on the mating, ovulation and external sexual characteristics [10–18]. As for the study of gonadal development, most of them are on simple morphological observation [12, 19–22]. While an early histological studies on the gonads of *L*. *seticaudata* at the male stage showed ovotestis composing of a posterior testicular and an anterior ovarian zone [23] and in *L*. *amhoinensis*, vitellogenic oocytes and mature sperm were observed in all individuals examined [12]; these studies were all based on specimens collected from the wild. As a result, there is no study so far tracking ontogenetic gonadal development from newly settled juveniles until they reached euhermaphrodite phase for a *Lysmata* species.

The peppermint shrimp *Lysmata vittata* is a small species from the genus *Lysmata*, which can grow to about 3 cm in total length. Its natural distribution is in the indo-pacific region, including the coastal waters of China, Japan and the Philippines [6, 24, 25]. However, it was recently collected from Egyptian Mediterranean Sea, and was speculated having been introduced to there with the ballast water of cargo ships [24, 26]. *L*. *vittata* is commonly found at a depth of 2–50 m and they are reportedly hiding in the shadow of the reef during the day but active during the night [27]. While Yang & Kim [28] have recently described larval development of the species, which include nine zoeal stages, based on laboratory-reared specimens; there is a lack of knowledge on its general reproductive biology, such as ontogenetic development of gonads and external sexual characters, which is necessary for providing baseline information for the study of the molecular mechanism of the unique sexual system of PSH.

## Materials and methods

*L*. *vittata* used for this study were captive bred at Fisheries Research Institute of Fujian Province, China located in Xiamen city. The shrimps were transported to the laboratory of Xiamen University, Fujian, China and acclimated in salt water aquaria (salinity: 34±1 ppt; water temperature: 28±1°C) when they were fed a commercial formulated diet daily. The shrimps were sampled continuously from early juveniles to the sexual maturity as euhermaphrodite individuals (carapace length ranged from 1.8 to 8.5 mm) for the study of ontogenetic gonadal and external sexual characters development.

When a specimen was sampled, it was firstly weighted, and then under a stereoscopic microscope, its carapace length, which is defined as the distance from the posterior edge of the eye orbit to the middorsal posterior edge of the carapace, was measured. The presence or absence of cincinnuli (hooks) on the endopod of the first pleopod, the presence of the appendix masculina of the second pleopod, and the location of the female and male gonopore opening, were observed and noted.

The shrimps were dissected after anesthesia on ice for 10 minutes. The gonads of the shrimps sampled were then dissected under a stereoscopic microscope to observe, record and take photos of morphology. The gonads were then fixed in modified Bouin’s fixative (1 L contains 250 ml 37–40% formaldehyde, 750 ml saturation picric acid and 5 ml glacial acetic acid) at 4°C for 24 hours and transferred to a 70% ethanol solution. The dehydration was carried out using an ascending sequence (70–100%) of ethyl alcohol, and then, the material was included in paraffin. Tissues were sectioned with a thickness of 6 μm and staining with hematoxylin and eosin (H & E) for histological observation. The study does not involve endangered or protected species.

## Results

### 1. Ontogenetic gonadal development

#### 1.1 Morphological characteristics of gonadal development

**Fig 1:**
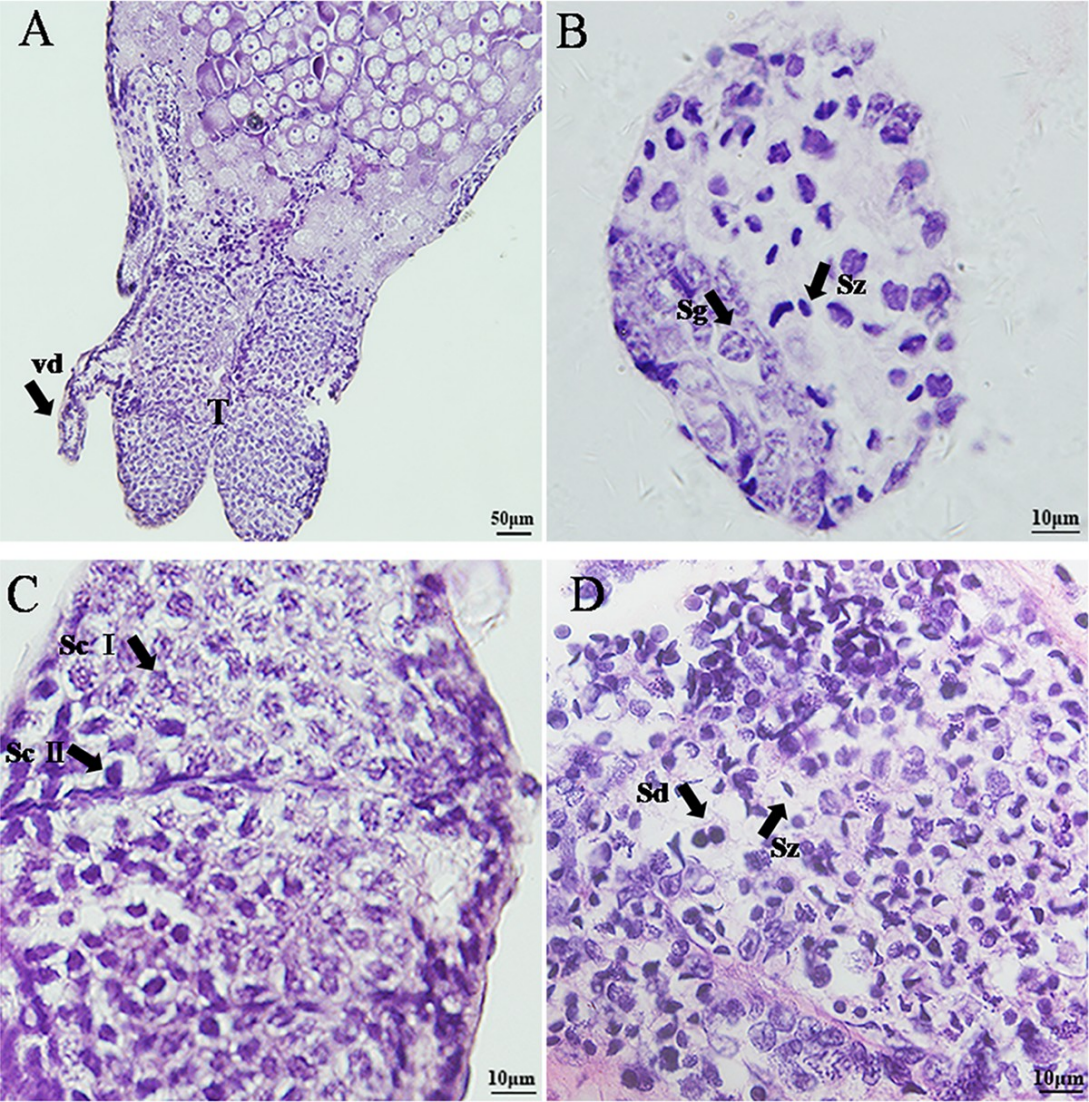
Histological characteristics of ontogenetic development of the testicular part of the gonad of *L*. *vittata*. A. ovotestis of Stage II; B. Stage I, the testicular part was mainly filled with spermatogonia, though different types of spermatogenic cells were found as well; C. Stage II, there were mainly spermatocytes I and spermatocytes II presented in the testicular region of the gonad; D. Stage III and IV, mainly spermatid and spermatozoa were found in the testicular region of the gonad. sg: spermatogonia; sd: spermatid; sc I: spermatocytes I; sc II: spermatocytes II; sz: spermatozoa; T: testis; vd: vas deferens.

**Fig 2:**
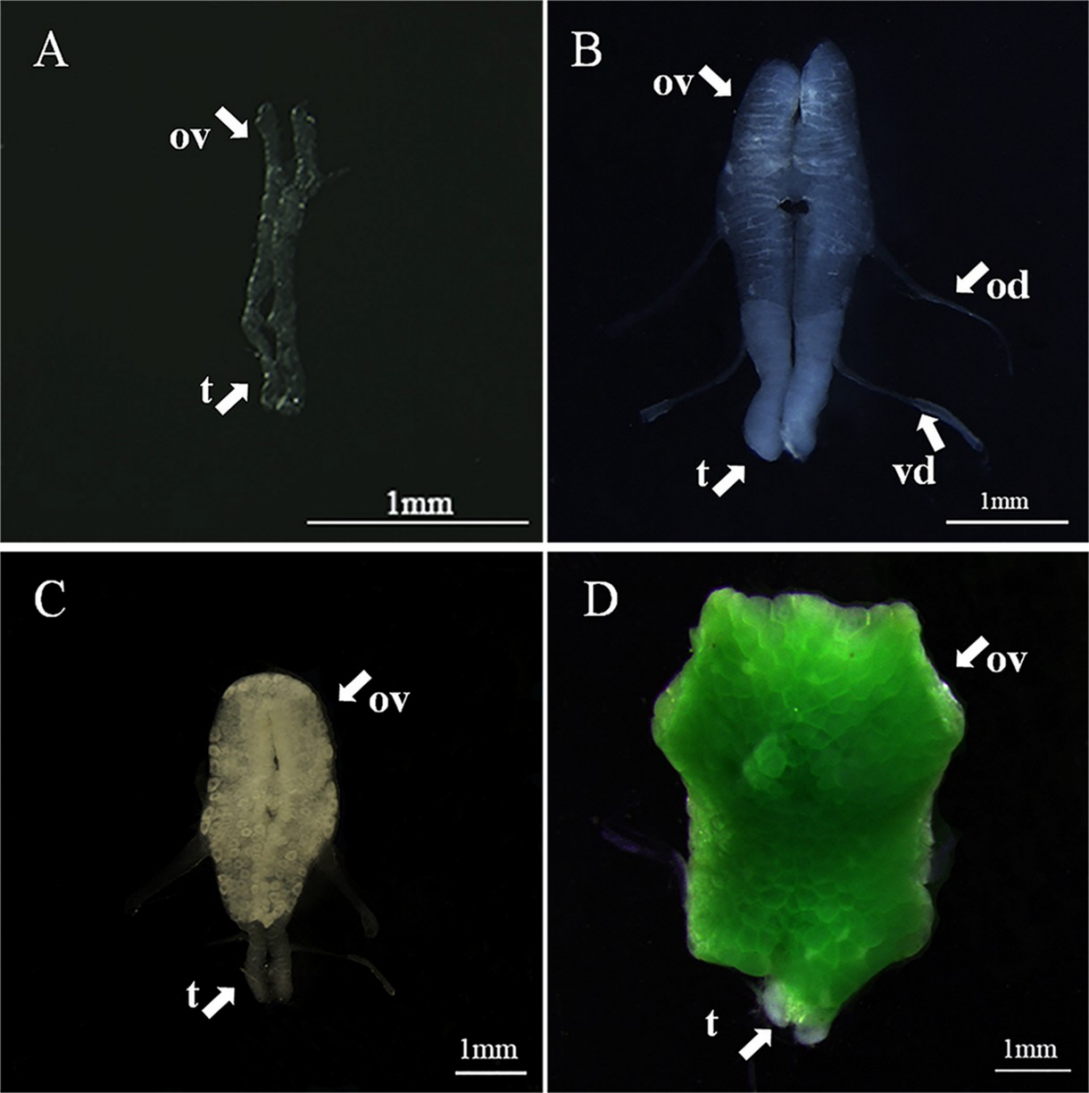
Morphological characteristics of gonadal development stages of *L*. *vittata*. A. Stage I, the gonad was transparent and small in size and the testicular part was substantially bigger than ovarian part, with inconspicuous oviduct and vas deferens; B. Stage II, the color of the gonad was cloudy white and the size of testicular part and ovarian part were roughly similar, with obvious oviduct and vas deferens; C. Stage III, the ovarian part was earthy brown or yellow-green color and the ovarian part was greater than the testicular part; D. Stage IV, the ovarian part was dark green and the testicular part in the gonad was much smaller than the ovarian part; ov: ovary; t: testis; od: oviduct; vd; vas deferens.

The gonad of *L*. *vittata* was H-shaped and located in the cephalothorax but extended to the junction of cephalothorax and abdomen. The anterior portion of the gonad was above the hepatopancreas and extended backward to the top of heart. All individuals examined showed ovotestes, which consisted of an anterior ovarian part and a posterior testicular part (Fig 1A). The ovarian region was formed by two lobes with a small gap in the middle. On the posterior of ovary, there was a pair of lateral oviduct (Fig 2B) that had openings at the base of the third pair of pereiopods to form female gonopores. In the anterior testicular region, there were two lobules with a lateral sperm duct in each of them, the sperm ducts opened at the base of the fifth pair of pereiopods to form male gonopores (Fig 2B). With the growth and development of the shrimps, their gonad morphology changed ontogenetically. Based on both morphological and histological evidence, four gonadal stages were determined for *L*. *vittata*, which is described in the following. From Stage I to III, the testicular part of the gonad had become gradually mature while the ovarian part was still immature, which was defined as the male phase. At Stage IV, both testicular part and ovarian part were mature, and therefore was defined as euhermaphrodite phase.

Stage I: found in shrimps with carapace length between 1.8–4.1 mm, including newly settled juveniles. At this stage, the gonad was transparent and small in size (approximately 1mm in length), making it difficult to dissect. The ovarian part was undeveloped and the proportion of the testicular part was substantially greater than ovarian part, with inconspicuous oviduct and vas deferens (Fig 2A).

Stage II: found in shrimps with carapace length between 3.7–5.5 mm. The color of the gonad changed from transparent to cloudy white, with size increased to approximately 3mm length. The size of testicular part and ovarian part were roughly similar, with obvious oviduct and vas deferens (Fig 2B).

Stage III: found in shrimps with carapace length between 4.1–7.5 mm. At this stage, the gonad length was about 5 mm, and in earthy brown or yellow-green color. The ovarian part was greater than testicular part, and extended forward (Fig 2C).

Stage IV: found in shrimps with carapace length between 5.8–8.5mm. The ovarian part was dark green and significantly enlarged, with the front of the ovary reaching almost to the base of the eyestalk at full maturity. At this time, the testicular part in the gonad was much smaller than the ovarian part, showing a tendency to degenerate (Fig 2D).

The major morphological characteristics of the gonadal development stages of *L*. *vittata* are summarized in Table 1.

**Table 1:** The major morphological characteristics of the gonadal development stages of *L*. *vittata*.

		Ovotestes	
Phases	Stages	Testicular region	Ovarian region
Male	I	Length> ovary; transparent	Length< testis; transparent
	II	~1/2 of gonad length; cloudy white	~1/2 of gonad length; cloudy white
	III	Length< ovary; cloudy white	Length> testis; earthy brown/yellow-green
Euhermaphrodite	IV	Length<<< ovary; cloudy white	Length>>> testis; dark-green

#### 1.2 Histological characteristics of gonadal development

**Fig 3:**
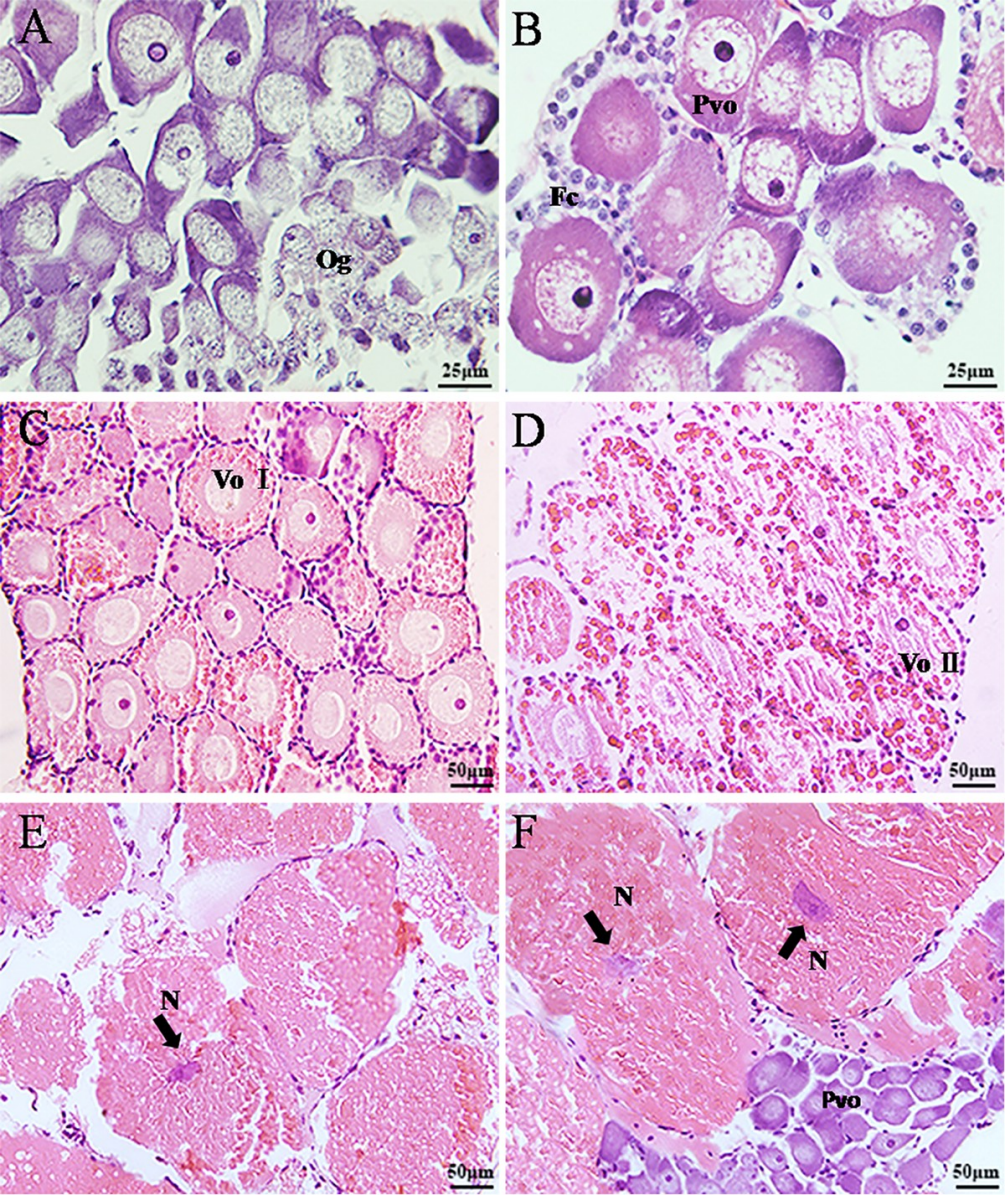
Histological characteristics of ontogenetic development of the ovarian part of gonad of *L*. *vittata*. A. Stage I, mainly oogonia and previtellogenic oocytes were presented in the ovarian part of the gonad; B. Stage II, mainly oval previtellogenic oocytes in the ovary; C. early Stage III, mainly vitellogenic I oocytes were found in the ovarian region; D. late Stage III, mainly vitellogenic II oocytes were found in the ovarian region; E. Stage IV, many mature oocytes were found in the ovarian region of the gonad; F. Stage IV, the previtellogenic oocytes were also observed at this stage sometimes; Og: oogonia; Pvo: previtellogenic oocytes; VoI: vitellogenic I; VoII: vitellogenic II; Fc: follicular cell; N: nucleus.

Stage I: at this stage, the testicular part of the gonad was mainly filled with spermatogonia, though different types of spermatogenic cells were found as well. Spermatogonia were round in shape, with a mean diameter of 8 μm and scarce cytoplasm and evenly distributed lumped chromatin (Fig 1B). Meanwhile in the ovarian part of the gonad, mainly oogonia and previtellogenic oocytes were presented. Oogonia were small, either round or suborbicular with a mean diameter of 15 μm. They possessed a large nucleus with scarce cytoplasm, which forms a thin layer surrounding the nucleus (Fig 3A).

Stage II: there were mainly spermatocytes I and spermatocytes II presented in the testicular region of the gonad. Compared to spermatogonia, spermatocytes I were slightly larger and nearly round with a mean diameter of 10 μm. The chromatin of spermatocyte I was scattered and started to condense. Smaller than spermatocyte I, spermatocyte II was deeply stained; and at the end of the first meiosis, chromatin halved and distributed toward one side (Fig 1C). On the other hand, in the ovary, there were mainly oval previtellogenic oocytes presented, which had more cytoplasm than the oogonia. The previtellogenic oocytes were basophilic and stained in blue-violet with prominent nucleolus. Several small vacuoles were observed in the cytoplasm, and filose or granular chromatin with scatter distribution. There were also a few follicular cells distribute among the oocytes (Fig 3B).

Stage III: at this stage, mainly spermatid and spermatozoa were found in the testicular region of the gonad. Spermatids were small with round nucleus, which were evenly stained and had a diameter of about 5 μm. As spermatid transforming to spermatozoa, the nucleus gradually moved to one side of the cells. Spermatozoa had crescent nucleus, making them easy recognizable (Fig 1D). Mainly vitellogenic oocytes were found in the ovarian region of the gonad, which grew fast in size due to rapid accumulation of yolk granules in the cytoplasm. This stage can be further divided into vitellogenic I and vitellogenic II stage. The nucleus of the vitellogenic I oocytes (60–120 μm) were large with distinct membrane, and nucleoplasm diffused in the nucleus. There were yolk granules in the cytoplasm and stained red (Fig 3C). The vitellogenic II oocytes (130–200 μm) continued to increase in size; and large yolk granules were densely found in the cytoplasm while some of them fused (Fig 3D). At this stage, each oocyte was found surrounded by a layer of elliptic follicular cells (Fig 3C and 3D).

Stage IV: at this stage, the spermatogenic cells in the testicular part were similar to those of stage III. Many mature oocytes were found in the ovarian region of the gonad with diameter 190–250 μm. A large quantity of yolk granules fused into slices, occupying the whole cytoplasm (Fig 3E and 3F). The nucleus began to shrink and stained in blue by H & E; the nuclear membrane started to disintegrate. The follicular cells appeared flatter as compared those at stage III. Besides the mature oocytes, the previtellogenic oocytes were sometimes also observed (Fig 3F).

### 2. External sexual characters

**Fig 4:**
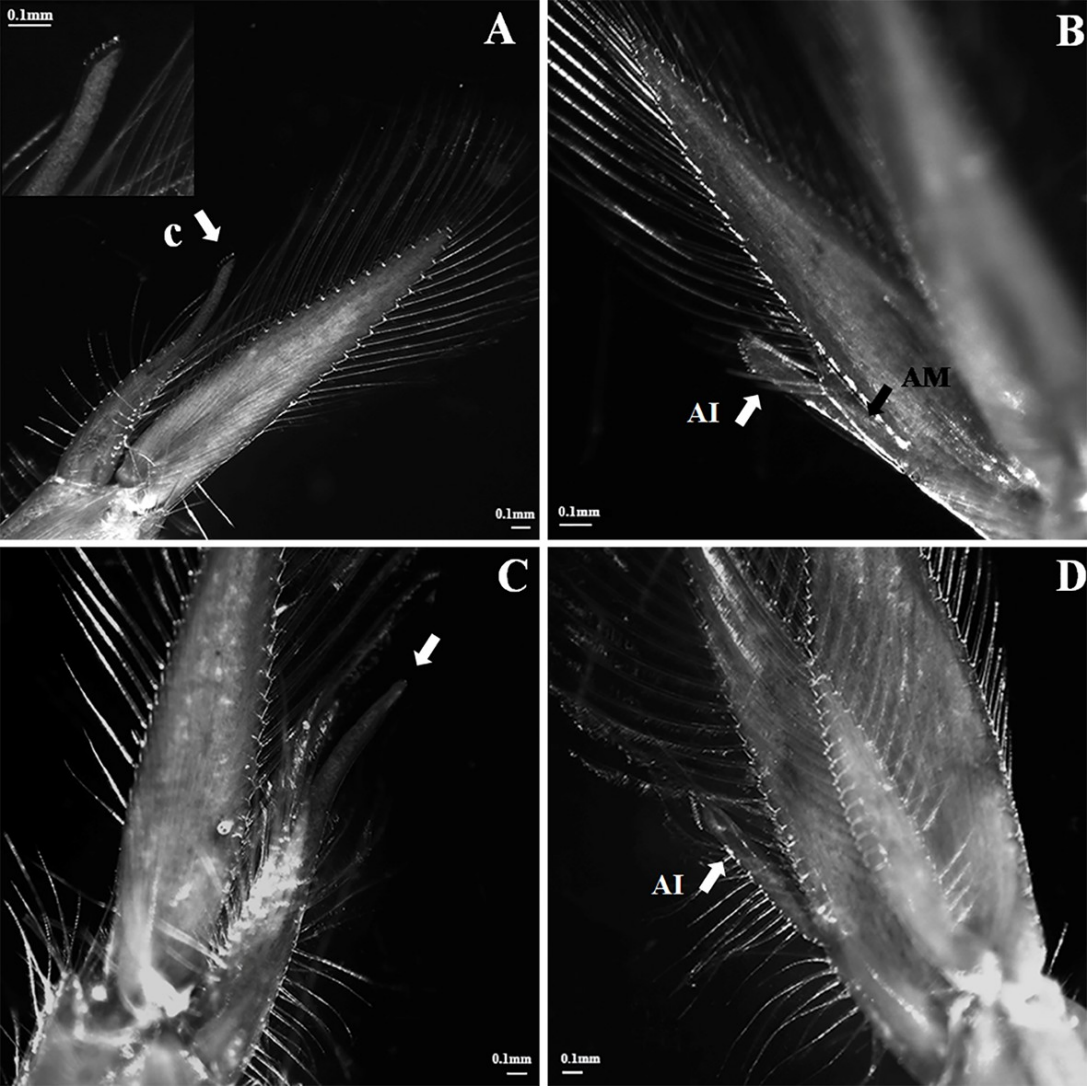
The first and second pleopods of *L*. *vittata*. **A & B: male phase; C&D: euhermaphroditic phase**. A. cincinnuli at the tips of the appendix interna on the first pair of pleopods; B. appendices masculinae (black arrow) on the second pair of pleopods; C. lack of cincinnuli at the tips of the appendix interna on the first pair of pleopods; D. lack of appendix masculina on the second pair of pleopods; c: cincinnuli; AM: appendices masculinae; AI: appendix interna.

Male phase: cincinulli (5–8 hooks) presented at the tips of the appendix interna on the first pair of pleopods (Fig 4A) while appendices masculinae (AM), in a form of a stick structure with spines, presented at the inner edge of the appendix interna (AI) on the second pair of pleopods (Fig 4B). The testicular part of the gonad was fully developed but the ovarian part was still not mature.

Euhermaphrodite phase: most individuals lack of cincinulli and appendices masculinae on the first and second pair of pleopods respectively (Fig 4C). On the other hand, there are many spines on the AI (Fig 4D). Both testicular and ovarian parts in the gonad were mature.

**Fig 5:**
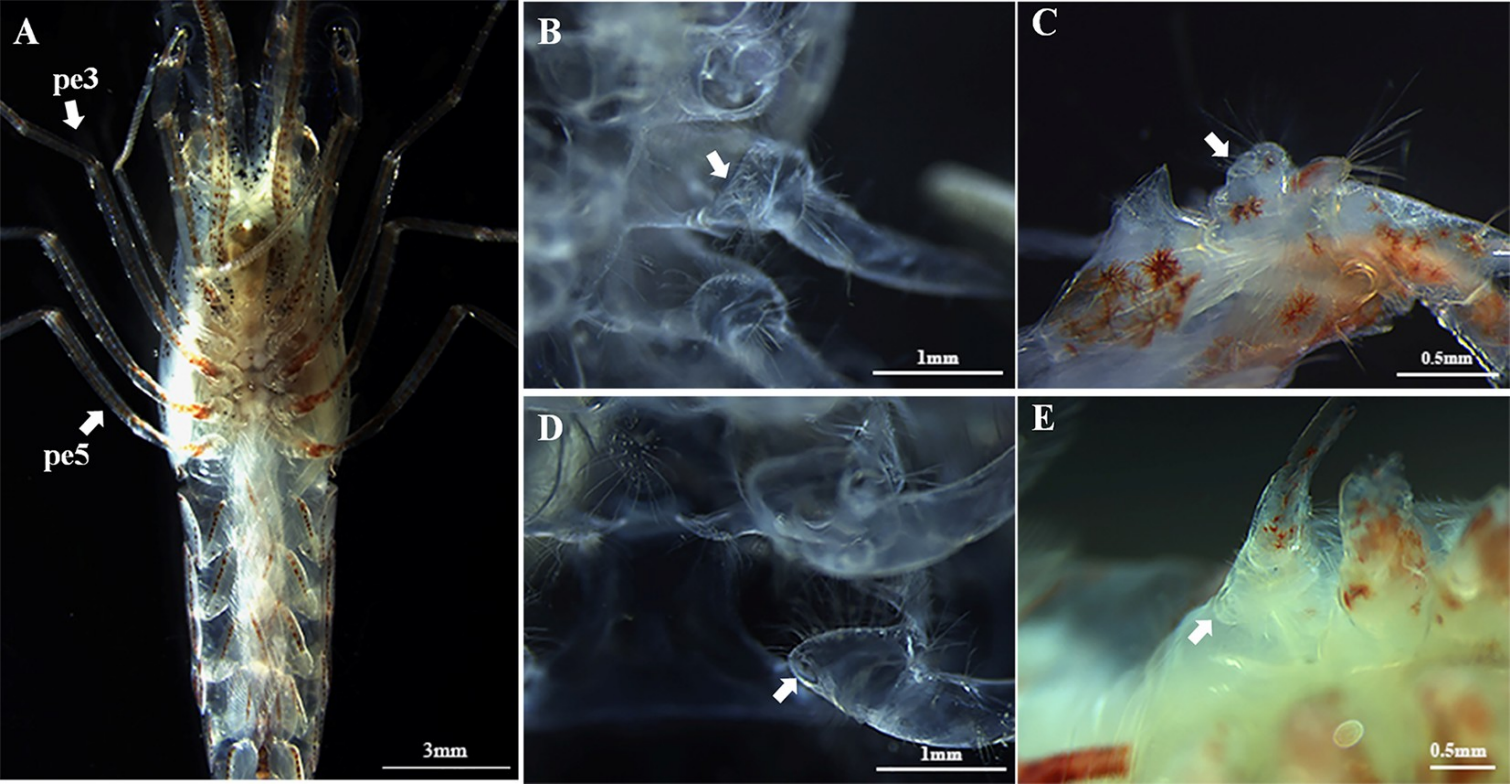
The location of male and female gonopores of *L*. *vittata*. A. The arrow indicates the third and fifth pereopod respectively; B. Female gonopore (arrow) at the base of the third pereiopod as shown on an exuvia; C. Female gonopore (arrow) at the base of the third pereopod; D. Male gonopore (arrow) at the base of the fifth pereiopodas shown on an exuvia; E. Male gonopore (arrow) at the base of the fifth pereiopod; pe3: the third pereiopod; pe5: the fifth pereiopod.

At both the male and the euhermaphrodite phase, female and male gonopores were found located on the coxa of the third (Fig 5A, 5B and 5C) and the fifth pair of pereiopods (Fig 5A, 5D and 5E), respectively.

Based on morphological characteristics of gonadal developmental stage mentioned above, all specimens examined (carapace length: 1.8–8.5 mm; body weight: 8–529 mg), including newly settled juveniles, were determined for their gonadal stages (Table 2). The length of the appendices masculinae (AM) was found related to the carapace length, weight and gonad developmental stage of the shrimps (Table 2). Based on their length, AM of all specimens examined were divided into three size classes: S (small): < 0.3 mm; M (medium): 0.3–0.5 mm and L (large): > 0.5 mm, and the quantity of each size class within each gonadal stage was calculated. At the male phase, AM length showed an increasing trend with the development of gonad and the increase of the carapace length (Table 2). At euhermaphrodite phase, most individuals lack of AM although in a few individuals, AM could still be found but have the appendix masculina with the degenerated.

**Table 2:** Carapace length, body weight, gonadal development stage and appendices masculinae length of *L*. *vittata*.

					Appendices masculinae			
Phases	Stages	n	CL (mm)	BW (mg)	lack	S(<0.3mm)	M(0.3–0.5mm)	L(>0.5mm)
Male	I	21	1.8–4.1	8.0–105.3	0	16	5	0
	II	24	3.7–5.5	80.2–217.3	0	3	19	2
	III	23	4.1–7.5	177.8–548.0	3	1	7	12
Euhermaphrodite	IV	23	5.8–8.5	245.5–601.5	21	2	0	0

Note: CL: carapace length; BW: body weight. L: large; M: medium; S: small.

## Discussion

Despite the unique sexual system of PSH is widespread in species from the genus *Lysmata* [13], full ontogenetic development of the gonad from newly settled juveniles to euhermaphrodite individuals has not be described previously. Such knowledge is important as baseline information for further study of this unique sexual system in crustaceans and its underlying molecular regulating mechanisms. To fill this knowledge gap, in the present study, based on captive bred specimens, ontogenetic gonadal development of *L*. *vittata* were studied both morphologically and histologically from newly settled juveniles.

The results showed that *L*. *vittata* is euhermaphrodite with an ovotestis composing of a posterior testicular and an anterior ovarian region found throughout their life cycle since newly settled juveniles. According to the characteristic of gonadal development, four gonadal stages were determined for *L*. *vittata*. Surprisingly, a small amount of spermatozoa were observed in the testicular part of the Stage I gonad within a few days of larval settlement as juveniles. At the Stage III, the testicual part was fully mature with a large quantity of mature sperms with cup-shaped nucleusas reported in typical Caridean species, such as *Macrobrachium amazonicum*, *M*. *rosenbergii* and *Exopalaemon carinicauda* [29–31]. There is a slight deterioration of the testicular region at the Stage IV, which may be related to mating occurred at the male phase. In other decapod crustaceans, such as *M*. *asperulum* and *Scylla paramamosain*, the testis in males reportedly also atrophied or degenerated after mating [32, 33].

The size and color of ovarian region of the gonads changed significantly with the growth of *L*. *vittata*, meanwhile the oogenesis encompassed five stages of oogonia, previtellogenic oocytes, vitellogenic I oocytes, vitellogenic II oocytes and finally as mature oocytes. Over the five stages of oogenesis, the ovarian color changed correspondingly form transparent to cloudy white, from cloudy white to earthy brown, and then from earthy brown to yellow-green, and finally from yellow-green to dark green, which might be used as a rough guide for estimating the advance of oogenesis. For example, when the ovarian part appeared earthy brown and yellow-green, oocytes are expected to be at the stages of vitellogenic I and vitellogenic II, respectively.

Although there were three past studies on histological features of the gonadal development of species from the genus *Lysmata* and *Exhippolysmata*, they were all based on wild collected specimens and lack of some important details, for instance, both studies did not report morphological characteristics of mature oocytes [12, 23, 34]. In the present study, *L*. *vittata* used were captive bred, therefore experimental animals at all stages of development could be obtained in sufficient number, which enabled continuous observation on ontogenetic gonad development from as early as newly settled juveniles as well as detailed study at any stage. Based on this study, as entering the middle stage of the first division of meiosis, the nucleus of mature oocytes of *L*. *vittata* started to shrink with the gradual disintegration of nuclear membrane. Such a character of mature oocytes is similar to that of other decapods, such as *Panulirus japonicus*, *Litopenaeus vannamei* and *S*. *paramamosain* [35–37]. It was also observed that while the stage IV gonads were dominantly by mature oocytes, there were still some previtellogenic oocytes presented, which may be related to allowing rapid re-maturation of the ovaries after ovulation. In fact, such a phenomenon was observed in mature ovaries of crustaceans with consecutive spawning feature, such as *L*. *vannamei* and *Portunus pelagicus* [36, 38].

*L*. *vittata* showed significantly different external sexual characteristics between male phase and euhermaphroditic phase: at the male phase, the opening of the male gonopore at the base of the fifth pereopod, cincinnuli at the tips of the appendix interna on the first pair of pleopods, and appendices masculinae on the second pair of pleopods were observed. At the euhermaphroditic phase, while individuals still showed male gonopores, most of them lacked the appendix masculina; and a few individuals still had appendix masculina, it was substantially smaller and degenerated. The external sexual charateristics of *L*. *vittata* are very similar to those reported for other species of the genus *Lysmata*, such as *L*. *wurdemanni*, *L*. *californica* and *L*. *bahia* [11, 14, 19, 21]. While it is believed that the genus *Exhippolysmata* has the same sexual pattern as *Lysmata* [39], the ontogenetic changes in external sexual characteristics is somewhat different. For example, *E*. *oplophoroides* has no cincinnuli on the first pair of pleopods whether at the male or the euhermaphroditic phase; and with the development of gonad, appendix masculina were found diminished but retained at all times [13, 39].

Past studies have shown that the sexual systems of Caridean species are very complicated. For example, the gonad of *Pandalus platyceros* from the family Pandalidaeis in the form of an ovotestis and only the testicular elements are functional during the male phase; however, at the female phase, the testicular part degenerated while ovarian part has developed and matured [40, 41]. Among species of the Crangonidae, *Crangon crangon* population includes both individuals who reproduce as females throughout their life cycle and those who reproduce as males first but later as secondary females [13, 42]. *C*. *franciscorum* was initially assumed to be gonochorism but later proved to have the same sexual pattern as *C*. *crangon* [43]. In family Hippolytide, the population of *T*. *manningi* includes individuals who remain males throughout and individuals who later turn into females [2]. The sexual system of the genus *Lysmata* is well known to be very specific protandric simultaneous hermaphrodite (PSH), which means at the euhermaphrodite phase, the testicular and ovarian parts of the shrimps are both mature, and therefore they are capable of mating with other individuals as both male and female [11–13]. Histological results of this study showing individuals at the euhermaphroditic phase (Stage IV) had both mature oocytes and mature sperms in the ovarian and testicular region, respectively, confirm this. In *L*. *wurdemanni* and *L*. *californica*, ‘‘transitional” individuals, which showed external sexual characters of males but had vitellogenic oocytes in the ovarian region of their gonads, were reported [11, 14]. In this study, such individuals were also found at the gonad Stage III.

It has been suggested that for *L*. *wurdemanni* and probably other species from the genus *Lysmata*, not all male phase individuals may transform into the euhermaphroditic phase, and social interactions may play an important role in mediating the transformation [11]. However, in *L*. *bahia* and *L*. *intermedia*, there was no individual was observed to not transform into the euhermaphroditic phase [19]. In this study, we also observed that the ovarian parts of gonads matured with dark-green color in all shrimps with carapace length above 5.8 mm, suggesting no individual remained at the male phase above this size.

In summary, by studying ontogenetic gonad development of *L*. *vittata* from newly settled juvenile both morphologically and histologically, the present research provides important baseline information for further studies of the unique sexual system of protandric simultaneous hermaphrodite in crustaceans, including its underlying molecular mechanisms.

## Conclusions

Based on both morphological (e.g., size, color and shape) and histological features (e.g., oogenesis and spermatogenesis), four gonadal development stages were defined and described for *L*. *vittata*. From Stage I to III, the testicular part of the gonad became gradually mature but the ovarian part was still immature, which is defined as the male phase. At Stage IV, both the testicular part and the ovarian part were mature and hence is defined as euhermaphrodite phase.
